# Changes in thrombin generation and D-dimer concentrations in women injecting enoxaparin during pregnancy and the puerperium

**DOI:** 10.1186/s12884-014-0384-0

**Published:** 2014-11-19

**Authors:** Jignesh P Patel, Raj K Patel, Lara N Roberts, Michael S Marsh, Bruce Green, J Graham Davies, Roopen Arya

**Affiliations:** Department of Haematological Medicine, King’s Thrombosis Centre, King’s College Hospital NHS Foundation Trust, Denmark Hill, London, SE5 9RS UK; Institute of Pharmaceutical Science, King’s College London, London, UK; Department of Obstetrics and Gynaecology, King’s College Hospital NHS Foundation Trust, London, UK; Model Answers Pty Ltd, Brisbane, Australia

**Keywords:** Blood coagulation, D-dimers, Enoxaparin, Postpartum period, Pregnancy, Thrombin generation, Venous thromboembolism

## Abstract

**Background:**

It is well accepted that the gravid state is hypercoagulable and a significant cause of both maternal morbidity and mortality in the Western world. Although thrombin generation is reported to be increased in pregnant women, uncertainty exists on the pattern of thrombin generation change during this time. The aim of this study is to describe thrombin generation changes and D-dimer concentrations in women injecting enoxaparin during pregnancy the postnatal period.

**Methods:**

One hundred and twenty-three women injecting enoxaparin had their thrombin generation, as measured by Calibrated Automated Thombinography (CAT), repeatedly assayed during pregnancy, once in each trimester, at delivery and 8 weeks post-partum. Furthermore, to understand the impact enoxaparin has on D-dimer concentrations during pregnancy, D-dimer concentrations were measured monthly in the recruited women.

**Results:**

Thrombin generation was found to increase in the first trimester (mean endogenous thrombin potential (ETP): 1391 nmol/L.min), further increasing during the second trimester (mean ETP: 1757 nmol/L.min), after which it plateaued through to delivery, where it peaked (mean ETP: 1857 nmol/L.min) and then fell back at 8 weeks post-partum (ETP: 1293 nmol/L.min). In contrast D-dimer concentrations increased exponentially during the antenatal period, despite the enoxaparin prescription.

**Conclusion:**

Our results provide further evidence on alterations of thrombin generation during pregnancy and the postnatal period.

**Electronic supplementary material:**

The online version of this article (doi:10.1186/s12884-014-0384-0) contains supplementary material, which is available to authorized users.

## Background

Global coagulation assays, such as the thrombin generation assay, explore the initiation, propagation and termination phases of coagulation [1], and so can detect an underlying prothrombotic state. Studies have demonstrated the utility of the thrombin generation assay in identifying the prothrombotic states seen with increasing age [2], in the obese [3], those with thrombophilia [4], users of oral contraception and hormone replacement therapy [5,6] and in those of African-Caribbean origin [7]. Only a small number of studies have explored thrombin generation during pregnancy [8-12]. Whilst the results of these studies demonstrate a hypercoagulable state, the pattern of thrombin generation changes during pregnancy has provided conflicting results. Knowing how thrombin generation is altered during the gravid period is important, as it could help define the periods of greatest risk and ultimately inform when and how interventions such as low molecular weight heparin (LMWH) are prescribed.

In contrast to the global thrombin generation assay, plasma concentrations of D-dimers represent a sensitive marker of active coagulation and subsequent fibrinolysis. Observational studies in the pregnant population have demonstrated D-dimer concentrations to increase in line with gestation [13-18]. Some studies have also demonstrated this to be the case when pregnant women concurrently inject prophylactic doses of LMWH [19,20].

The aim of this study was to investigate how thrombin generation and D-dimer concentrations were altered in pregnant women injecting enoxaparin during pregnancy and the postnatal period.

## Methods

### Study setting

King’s College Hospital (Denmark Hill site) is a 900-bed tertiary care hospital, with around 5,400 babies delivered each year, including 1,500 caesarean sections. Nulliparous and multiparous women prescribed antenatal enoxaparin were eligible for entry to the study. Women who did not wish to participate or were unlikely to comply with directions, those with impaired renal function and those <18 years of age were excluded. Following informed written consent, subjects attended the thrombophilia clinic monthly during their pregnancy for ongoing monitoring, as well as drawing blood samples for determination of renal and liver function, full blood count, and D-dimer concentrations. Once in each trimester, women had additional blood drawn for the assessment of thrombin generation. Samples for assessment of D-dimer and thrombin generation samples were also drawn at the time of delivery and at ≥8 weeks post-partum; the latter sample providing a baseline thrombin generation and D-dimer concentration for subjects, acting as their own control.

### Sample collection and plasma preparation: D-dimers

Samples were obtained through antecubital venepuncture using a butterfly Terumo® Surflo® winged infusion set (21G × ¾”, 0.8 × 19 mm UTW tubing, REF: SV-21BL, Terumo Europe N.V., Belgium). For determination of D-dimer, 2.7 mL blood sample was collected in 0.109 M (3.2% trisodium citrate) Becton-Dickinson Vacutainer®, centrifuged at 2500 g for 7 minutes on a Rotina 38 R centrifuge (Hettich Zentrifugen), within 1 hour of collection. The resultant plasma was analysed using the STA-Liatest® D-DI kit (immune-turbidimetric assay of D-dimer (Diagnostica Stago, France)). Analysis was conducted using the STA-R evolution (Diagnostica Stago) analyser.

### Sample collection and plasma preparation: thrombin generation

Samples were obtained through antecubital venepucture using the aforementioned infusion set, without the use of a vacuum and with minimal suction into a 20 ml syringe, with the first 5 ml of blood drawn discarded. The blood was then transferred to 0.109 M (3.2% trisodium citrate) Becton-Dickinson Vacutainer®. Platelet poor plasma (PPP) was obtained by centrifugation at 4750 g for 10 minutes at room temperature. The supernatant was decanted into a polypropylene tube and centrifugation repeated. The plasma was decanted into a plastic tube, capped and immediately frozen. This procedure was performed within 60 minutes of venepuncture. The samples were stored at −35°C and analysed using calibrated automated thrombinography (CAT), as developed by Hemker [21].

### Calibrated automated thrombinography

Thrombin generation was measured with the thrombinoscope assay (Thrombinoscope BV, The Netherlands), according to the manufacturer’s instructions, as has previously been described by our group [6,7,22]. Concentrations of tissue factor and phospholipid used in the experiments were 5 pmol/L and 4 μmol/L, respectively.

Five thrombin generation parameters were computed: lag time, time to peak, peak height, start-tail and endogenous thrombin potential (ETP).

Intra- and inter-assay variability was assessed using plasma from a healthy control (male subject, aged 36–37 years). This volunteer was bled every eight weeks from the 16^th^ April 2010 to the 24^th^ January 2012. Eight plasma samples were obtained from the volunteer on each occasion and one sample was run in each thrombin generation experiment, from the 20^th^ April 2010 (exp 17) to the 21^st^ February 2012 (exp 125). For the primary thrombin generation parameters, lag-time, ETP, peak height, time to peak and start tail, the following coefficients of variation (%) were obtained: 4.5, 1.6, 3.9, 4.0, 2.1 for intra-assay variation and 7.6, 11.8, 13.7, 7.5 and 5.1 for inter-assay variation.

### Statistical analysis

Data was grouped into five time points: first, second and third trimester, within a week of delivery and ≥8 weeks post-partum.

Continuous variables are presented as means and standard deviations or median and inter-quartile ranges for normally and non-normally distributed variables respectively. For thrombin generation, differences in the means of variables were compared between the five time points, using the repeated measures ANOVA with a Greenhouse-Geisser correction. Post-hoc analysis was conducted using Bonferroni correction. For D-dimer concentrations, the Friedmans mean rank test was used to assess if statistically significant differences existed between the different time-points, with the Wilcoxon pair test used to assess significance between specific pairs of study time points.

As the Caucasian and African-Caribbean ethnic groups comprised the majority of the subjects recruited, differences in the thrombin generation and D-dimers results at each of the time points were compared for women injecting *prophylactic* doses of enoxaparin, using the independent *t*-test for thrombin generation and the Mann–Whitney U independent rank-sum test for D-dimer concentrations.

Further, to assess the impact of enoxaparin dose on the thrombin generation parameters and D-dimer concentrations, women were grouped into those injecting treatment doses and those injecting prophylactic doses. Descriptive statistics was used to report these results.

Statistical significance was considered at the 0.05 α level. Analysis was performed using SPSS version 18.0 (SPSS Inc., Chicago, Ilinois, USA).

The study received ethical approval from the Isle of White, Portsmouth and South East Hampshire Ethics Committee (REC reference: 09/H0501/57).

## Results

### Study participants and pregnancy outcomes

One hundred and nineteen patients had 123 pregnancies (4 women had two pregnancies during the study period). Five (4%) of the 123 pregnancies were twin pregnancies. The mean age of the recruited cohort was 33.11 years (range 18–46), and for 26 women, the index pregnancy was their first. A significant number of women recruited were obese class I-III (27 women (22%)). Table 1 summarises demographic information.

**Table 1:** **Demographic information on the recruited subjects and the indication for the enoxaparin prescription**

**Demographic**	**Number** ***N = 123***	**(%)**
*Ethnicity*		
Caucasian	66	(54)
African-Caribbean	37	(30)
Asian	9	(7)
Other	11	(9)
*Primi gravid*	26	(21)
*Body mass index (kg/m* ^*2*^ *)*		
Underweight (<18.5)	2	(2)
Normal (18.5-24.9)	62	(50)
Overweight (25–29.9)	32	(26)
Obese Class I (30–34.9)	7	(6)
Obese Class II (35–39.9)	4	(3)
Obese Class III (≥40)	16	(13)
*Indication for antenatal enoxaparin*		
Prophylaxis of VTE	83	(68)
Treatment of VTE	9	(7)
Antiphospholipid syndrome	9	(7)
Patients converting from long term warfarin	10	(8)
Other*	12	(10)

*7 patients with recurrent miscarriage in the absence of APLS, 1 patient with a history of retinal artery occlusion, 1 patient with a suspected patent foramen ovale, 1 patient with a history of a stillbirth – histology findings of the placenta reported a perivillous fibrin deposition, placental ageing and dysmaturity, 1 patient with a history of intrauterine growth retardation and abruption, 1 patient with a history of intrauterine growth retardation and pregnancy induced hypertension.

The range of enoxaparin doses prescribed for the subjects recruited are presented in Additional file 1: Table S1. Furthermore, details on the 19 women prescribed *treatment* doses of enoxaparin (9 women managed for antenatal VTE and the 10 women switched from long-term warfarin during pregnancy) are presented in Additional file 2: Table S2 and Additional file 3: TableS3 respectively.

### Pregnancy outcomes

Eight of the 123 pregnancies miscarried; 5 women (4.06%) had first trimester miscarriages and 2 women (1.62%) had second trimester miscarriages. The remaining patient had a still birth at 39 weeks gestation; One hundred and fourteen patients had 119 live births (delivery information on one woman was not available, as she emigrated prior to delivery). Table 2 presents delivery information on the study cohort.

**Table 2:** **Delivery outcomes in the recruited subjects**

	**Study population**
	***N = 114*** **(%)**
**Gestation delivered (weeks)**	38.8
**Blood loss recorded at delivery (ml)**	433.95
**Delivery Method** ***n*** **(%)**	
Vaginal	61 (54)
LSCS (elective)	23 (20)
LSCS (emergency)	20 (17)
Ventouse	6 (5)
Forceps	4 (4)
**Twin pregnancies**	5 (4)
**Gender**	
Boy	62 (52)
Girl	57 (48)
**Mean Baby weight (g)**	3162.43
APGAR score @ 1 minute (mean (min-max))	8.40 (0–10)
APGAR score @ 5 minutes (mean (min-max))	9.50 (0–10)

### Thrombin generation

The 123 subjects provided 498 individual thrombin generation profiles; 414 (83%) of the profiles were drawn from women prescribed *prophylactic* doses of enoxaparin with the remaining 84 profiles from women prescribed *treatment* doses.

Overall, the different parameters of the thrombogram were altered during pregnancy and the postpartum period (Table 3).

**Table 3:** **Overall thrombin generation results for the cohort of women recruited**

**TG parameter**	**1** ^**st**^ **trimester**	**2** ^**nd**^ **trimester**	**3** ^**rd**^ **trimester**	**Within 1 week of delivery**	**≥8 weeks pp**
	***N = 58; n = 44***	***N = 117; n = 94***	***N = 132; n = 108***	***N = 93; n = 93***	***N = 98; n = 98***
Lag time (min)	4.27 (3.81)	3.53 (1.26)	3.88 (2.46)	3.14 (1.32)	3.13 (1.34)
ETP (nmol/L.min)	1391 (795)	1757 (706)	1692 (631)	1857 (633)	1293 (409)
Peak height (nmol/L)	208 (141)	277 (136)	262 (116)	356 (127)	261 (80)
Time to Peak (min)	8.09 (5.18)	7.05 (3.27)	7.36 (3.91)	5.57 (2.83)	5.68 (1.58)
Start tail (min)	27.3 (9.7)	26.4 (6.9)	26.6 (6.8)	24.2 (5.8)	20.6 (2.79)

Mean with sd in brackets presented; pp refers to post-partum. *N* refers to the number of samples analysed at each time point, n refers to the number of women providing these samples.

The samples drawn at ≥8 weeks post-delivery provide an insight into the cohort of women’s *baseline* thrombin generation was prior to their pregnancy by which time-point women had discontinued their anticoagulation therapy. Using the ≥8 week post-partum values as a reference point; a rise in ETP is seen over the course of pregnancy, despite an enoxaparin prescription.

**Figure 1:**
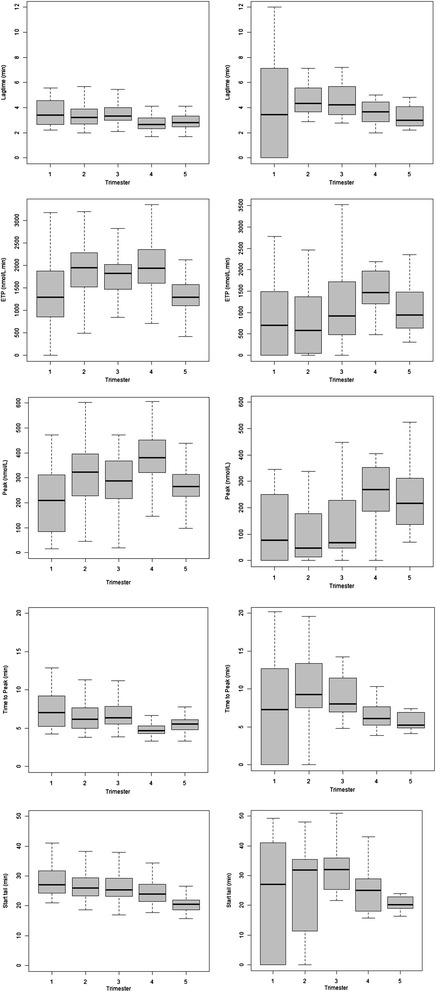
**Box plots illustrating the alterations in the five primary thrombin generation parameters for women injecting prophylactic (left), treatment (right) doses of enoxaparin during pregnancy and the post-partum period.** Trimester 4 refers to delivery and trimester 5 to ≥8 weeks post-partum.

When the thrombin generation results are separated according to women injecting treatment and prophylactic doses of enoxaparin, a difference in changes are observed between the group of women injecting treatment doses (84 profiles) and those injecting prophylactic doses (414 profiles) (Figure 1).

The results demonstrate a dose dependent enoxaparin effect. The ETP rise over pregnancy for women on treatment doses of enoxaparin is more attenuated, relative to those women prescribed prophylactic doses, as observed by differences in peak height. An influence of enoxaparin on the lag time, time to peak, and start tail is also observed. Examining the results from the women prescribed prophylactic doses, where all anticoagulant therapy had been discontinued by eight weeks postpartum, one can see that ETP is already higher by the first trimester, with a further rise seen during the second trimester, after which it settles through to delivery, where it peaks.

Eight women prescribed treatment doses of enoxaparin, had thrombin generation measurements at all study time-points. No significant difference in results for the individual thrombin generation parameters were found, across these five time-points. This was not the case, for women prescribed prophylactic doses of enoxaparin. In this group, 22 women had thrombin generation assayed at all study time-points. The following results were found for each thrombin generation parameter;

- Lag-time: significant differences were found between the time points (F(2.684, 56.261) = 3.732, *p* < 0.05). Post-hoc analysis demonstrated significant differences between the 8 weeks postpartum samples and the samples drawn in the first, second and third trimester, where a prolongation of the lag-time was observed.
- ETP: the mean ETP concentrations did statistically differ between the five time points (F(2.312, 48.561) = 9.072, *p* < 0.05). Post-hoc analysis revealed that specific differences were between the 8 weeks post-partum time point and the second and third trimesters and at the time of delivery, mirroring the result seen for the cohort of women as a whole.
- Peak height: statistically significant differences between the five time points (F(2.375, 49.877) = 5.209, p = <0.05) were found. A post-hoc analysis however, was unable to identify at which time points the differences were.
- Time to peak: significant differences were found (F(2.715, 57.021) = 6.181, p = <0.05). A post-hoc analysis revealed these differences between the ≥8 weeks post-partum time point and the first, second and third trimesters. No significant differences were found between the samples drawn at delivery and the ≥8 week post-partum time point.
- Start-tail: significant differences were found (F(2.252, 47.291) = 11.953, p = <0.05). A post-hoc analysis revealed that the specific differences were between the ≥8 weeks post-partum time point and the other four time points.

### Variation in thrombin generation by ethnicity

The Caucasian and African-Caribbean ethnic groups represented the majority of ethnic origin groups in the cohort of women recruited during the course of this study. The thrombin generation results from these two groups of women prescribed *prophylactic* doses of enoxaparin were also compared (Table 4).

**Table 4:** **Thrombin generation variation between the Caucasian and African-Caribbean groups**

**Parameter**	**Ethnicity**	**1** ^**st**^ **trimester**	**2** ^**nd**^ **trimester**	**3** ^**rd**^ **trimester**	**Within one week of delivery**	**≥ 8 weeks postpartum**
		***N*** **= 34 v 8**	***N*** **= 67 v 24**	***N*** **= 75 v 27**	***N*** **= 45 v 23**	***N*** **= 47 v 24**
Lag time (min)	Caucasian	4.11 (1.74)	3.49 (0.83)	3.62 (0.98)	3.03 (1.09)	2.98 (0.57)
	A-C	3.27 (0.81)	3.18 (0.74)	3.34 (0.77)	2.73 (0.61)	2.72 (0.51)
ETP (nmol/L.min)	Caucasian	1296 (651)*	1806 (557)*	1784 (451)	1944 (578)	1306 (356)
	A-C	2126 (754)	2129 (556)	1810 (432)	1911 (540)	1402 (336)
Peak height (nmol/L)	Caucasian	193 (124)*	284 (114)*	280 (92)	379 (119)	256 (60)
	A-C	323 (146)	356 (112)	301 (96)	367 (91)	286 (63)
tt Peak (min)	Caucasian	8.27 (3.6)	6.83 (1.8)*	6.95 (1.9)	5.23 (1.6)	5.65 (0.9)*
	A-C	6.61 (2.2)	5.89 (1.3)	6.22 (1.6)	4.88 (1.0)	5.15 (0.9)
Start tail (min)	Caucasian	27.94 (7.4)	26.37 (4.3)	26.07 (4.7)	24.29 (3.8)	20.42 (2.7)
	A-C	28.10 (3.1)	26.73 (3.9)	25.93 (4.2)	24.40 (4.1)	20.69 (2.8)

*Significance at <0.05 level; A-C refers to the African-Caribbean population, C refers to Caucasian population. *N* = number of samples in each trimester (Caucasian vs African-Caribbean). Mean with SD in brackets presented.

There are significant differences between the two ethnic groups for the mean ETP, and peak parameters with the African-Caribbean group exhibiting higher values for these relative to their Caucasian counterparts, during the first and second trimesters.

### D-dimers concentrations

Subjects provided 815 separate D-dimer concentrations from their pregnancies and the puerperium. Table 5 lists the pattern of D-dimer concentrations change from each of the time-points during the study. D-dimer concentrations rose during pregnancy (315 ug/ml), peaking at the time of delivery (1497 ug/ml), and returning to ‘baseline’ concentrations by 8 weeks postpartum (335 ug/ml).

**Table 5:** **The overall D-dimer concentration results for the recruited women**

**Pregnancy time point**	***N***	**Median (ug/mL)**	**Inter-quartile range**
First trimester (53)	88	315	245-467
Second trimester (103)	240	620	450-970
Third trimester (113)	287	990	690-1550
Within a week of delivery (102)	102	1497	2225-3910
≥ 8 weeks postpartum (98)	98	335	230-482

*N* represents the number of D-dimer samples at each study time point; numbers in brackets next to each time-point refer to the number of women providing samples at that time-point.

Although considerable inter-patient variability is exhibited, a clear pattern was observed in line with gestational age.

Significant differences were found when each study time-point was compared, except when the first trimester was compared with the ≥8 weeks post-partum time point.

### Women injecting prophylactic versus treatment doses of enoxaparin: D-dimer concentrations

Table 6 describes the D-dimer concentrations for women receiving prophylactic doses of enoxaparin (619 concentrations), compared to women injecting treatment doses (196 concentrations).

**Table 6:** **D-dimer concentrations (ug/ml) for the cohort of women prescribed prophylactic versus treatment doses**

**Pregnancy time point**	**Prophylactic dose prescribed**		**Treatment dose prescribed**	
	***N***	**Median concentration in ug/ml (inter-quartile range)**	***N***	**Median concentration in ug/ml (inter-quartile range)**
First trimester	64	335	24	270
		(270–487)		(230–422)
Second trimester	193	620	47	480
		(465–955)		(350–1180)
Third trimester	225	1020	62	850
		(735–1625)		(545–1270)
Within a week of delivery	79	2360	23	1610
		(1790–4130)		(440–2450)
≥ 8 weeks postpartum	78	365	20	260
		(256–525)		(220–325)

*N* represents the number of D-dimer samples at each time point.

Twenty-six women injecting prophylactic doses of enoxaparin had serial D-dimer concentrations measured consecutively from the first trimester through to ≥8 weeks post-partum. Statistically significant differences between the five different time points were found (n = 26, df = 4, p = <0.05). When the different study time points were compared with one another, significant differences were found between each study pair, except when the first trimester was compared with the ≥8 weeks post-partum time point.

Ten women prescribed treatment doses of enoxaparin had serial D-dimer concentrations consecutively measured from the first trimester through to ≥8 weeks of delivery. Statistically significant differences were found between the five study time points (n = 10, df = 4, p = <0.05); specifically between the second and third trimesters and within a week of delivery and ≥8 weeks postpartum.

When the Caucasians and African-Caribbean women injecting prophylactic enoxaparin were compared, no significant differences in D-dimer concentrations between these two groups at all-time points were found (Table 7).

**Table 7:** **Median D-dimer concentrations (ug/ml) for the Caucasian and African-Caribbean ethnic groups of women prescribed prophylactic doses of enoxaparin**

**Time point**	**Ethnicity**		***p-value*** *
	**Caucasian**	**African – Caribbean**	
First Trimester	340	335	U = 247, p = 0.925
	(267–512)	(302–430)	
Second Trimester	600	665	U = 2709, p = 0.225
	(457–865)	(492–992)	
Third Trimester	1000	1135	U = 3541, p = 0.071
	(715–1540)	(877–1687)	
Within a week of delivery	2400	2350	U = 505, p = 0.760
	(1690–4250)	(1760–4610)	
≥ 8 weeks post-partum	365	350	U = 504, p = 0.750
	(230–505)	(300–490)	

Median (interquartile range) D-dimer concentrations presented; *Mann–Whitney U independent rank-sum test.

## Discussion

To the best of our knowledge, our study is the largest to date, evaluating thrombin generation in pregnant women concurrently injecting enoxaparin, involving 498 separate thrombin generation profiles. This study is one of only two, to measure thrombin generation repeatedly in the same women during their pregnancy and the only study to couple this with thrombin generation measurements at the time of delivery and 8 weeks post-partum.

The primary aim of this study, was to describe how thrombin generation, as measured by CAT, was altered during pregnancy in the cohort of women recruited. With the 8 weeks post-partum sample acting as a baseline measurement for the recruited women, we found a rise in thrombin generation had already occurred in the first trimester, with a further rise during the second trimester, after which a thrombin generation plateau was reached through to delivery. At delivery, thrombin generation peaked and then dropped off by 8 weeks post-partum. This antenatal pattern of thrombin generation alteration is in agreement with the studies of Rosenkranz [9] and Dargaud [10]. Other studies provide differing descriptions of thrombin generation changes during pregnancy. The first to assess thrombin generation in this setting was Eichinger and colleagues [23]. In their study, thrombin generation was measured at fixed time-points (12, 22, 34 weeks gestation and 3 months post-partum). They used a tissue factor concentration of 120 pM in their experiments and report thrombin generation not to increase during pregnancy, despite evidence of substantial activation of the fibrinolytic system, through assessment of Fragment 1 + 2, Thrombin-Antithrombin complex and D-dimer concentrations. It could well be, that the high tissue factor concentration used in their study, was masking the true results, that have been reported here and by others. More recently McLean and colleagues have described thrombin generation in pregnant women, by measuring thrombin generation pre-conception, during early pregnancy (11–15 weeks), during late pregnancy (30–34 weeks) and again between 6–24 months after delivery [11]. The authors report thrombin generation increased during pregnancy in line with gestation. However, the authors did not measure thrombin generation during the second trimester, so are unable to conclude categorically from their results whether the increase continues during the second trimester or has reached a plateau by this time. More recently, Joly and colleagues [12] studied 101 women with uncomplicated pregnancies, and correlated thrombin generation, as measured by CAT with fibrinogen and other markers of blood coagulation activation. Only one blood sample was collected for each subject in this study, and they report thrombin generation to have increased early on during in pregnancy and then remains stable throughout the remainder of normal pregnancy. As the same women were not repeatedly tested, it is difficult to categorically draw this conclusion; indeed this limitation is apparent in many of the studies exploring thrombin generation during pregnancy and the puerperium.

The published research to date appears to agree that the prothrombotic state of pregnancy can be detected by the global coagulation assay, thrombin generation, and that this prothrombotic state is detected from the first trimester. What remains unclear is how, thrombin generation is altered through the remainder of pregnancy. When examining the distribution of VTE through pregnancy, some studies report a bimodal distribution of antenatal VTE with most events in the first and third trimester [24] and others suggest the incidence to increase non-significantly with increasing gestation [25]. Studies from the United Kingdom describing the incidence of VTE during pregnancy in recent years, describe the incidence to increase with gestation; Voke and colleagues study of antenatal VTE, reported 31 women presenting in the first trimester, 37 during the second trimester and 58 during the third trimester [26]. In Knight and colleagues study of antenatal PE, the median gestational age at diagnosis was 28 weeks gestation (range 2–40 weeks) [27].

In order to better understand the relationship between thrombin generation and the incidence of VTE during pregnancy, and whether an increased level of thrombin generation can predict an antenatal VTE event, there is clearly a need for a study which evaluates thrombin generation during uncomplicated pregnancy in the same women at pre-determined pregnancy time points, using standardised methods and reagents. Such a study, would not only resolve the uncertainty, it would help provide a comparator for studies evaluating thrombin generation changes in women with other antenatal complications, such as pre-eclampsia.

During our study, the presence of enoxaparin in the women’s plasma samples will of course be impacting on the results reported. However, the results suggest only a small impact of prophylactic enoxaparin on thrombin generation (as measured by CAT) and that this effect is unlikely to interfere with the pattern of thrombin generation being described here. We are relatively confident of this, in light of what others have also previously reported. Gerotziafas and colleagues [28] assessed the inhibition of thrombin generation induced by different LMWH *in-vitro*, in platelet rich plasma. They found that enoxaparin impact on thrombin generation at a concentration less than 0.2 IU/mL was minimal, when compared to unfractionated heparin and tinzaparin, and anti-Xa concentrations greater than 0.4 IU/mL were required to induce a significant reduction in the ETP and peak height. A similar finding has been reported by Adamidou [29]. Green and colleagues [30] assessed the thromboprophylatic effect of dalteparin or rivaroxaban in those patients who had undergone elective hip and knee replacements. Both these agents are effective in preventing VTE in this setting. They found patients who were prescribed dalteparin exhibited a variable thrombin generation response after surgery, whereas the effects of rivaroxaban were more consistent. Rivaroxaban was found to inhibit thrombin generation (as measured by CAT) more, in relative terms, than dalteparin at 24 hours post-surgery, even though it is accepted that they are equally efficacious. The results considered together seem to suggest that the full impact of prophylactic doses of enoxaparin are not borne out in the thrombin generation assay.

### D-dimer concentrations

D-dimer concentrations reflect active coagulation and fibrinolysis. Our large set of D-dimer results agree with previously published studies, that D-dimer concentrations increase exponentially during pregnancy, peaking at the time of delivery. To date, little work has been published on how the D-dimer concentrations might be altered in pregnant women who are concurrently injecting LMWH. Hoke and colleagues have previously explored this [19] and assessed 61 women prescribed LMWH thromboprophylaxis throughout pregnancy due to a history of VTE, hereditary thrombophilia and/or previous pregnancy related complications. D-dimer concentrations were measured during their pregnancy, having been prescribed dalteparin or enoxaparin at a dose range of 4000 to 7500 anti-factor Xa IU daily; commenced following a positive pregnancy test. The D-dimer concentrations were then compared with a control group comprising 113 healthy pregnant women not requiring thromboprophylaxis. The author’s found that D-dimer concentrations in both subjects and controls increased significantly from the first to the second trimester, as well as from the second to the third trimester. The authors also describe the D-dimer concentrations being significantly higher among subjects compared with controls. Despite being prescribed prophylactic LMWH, substantial activation of coagulation was seen during pregnancy in this study, i.e. the prophylactic doses of LMWH did not dampen down the increase in D-dimers one might expect to observe. Furthermore, Abou-Nassar and colleagues [20], as part of the Thrombophilia in Pregnancy Prophylaxis Study (TIPPS), patients at high risk of pregnancy complications with confirmed thrombophilia were randomised to receive either dalteparin 5000 units daily until 20 weeks gestation, then 5000 units twice a day until 37 weeks gestation or onset of labour, or no treatment. Samples for assessment of TAT, prothrombin fragment 1 + 2, and D-dimer concentrations were drawn in each trimester. The authors report that the levels of TAT, prothrombin fragment 1 + 2 and D-dimer concentrations increased significantly in the dalteparin and control group, again illustrating that the prophylactic LMWH does not impact on the D-dimer concentrations. The results from our study confer with the findings from both these studies.

The increase in D-dimer concentrations during pregnancy, appear relatively predictable and the utility of using D-dimer concentrations for the diagnosis of antenatal VTE should be explored further, with a view of developing a *normal* D-dimer pregnancy reference range, as has been suggested previously [31-33]. The UK National Institute for Health Research recently made a Health Technology Assessment call for researchers to define the optimal algorithm to diagnose antenatal pulmonary embolism. The results from this commissioned research [34] will be eagerly awaited in years to come and perhaps D-dimer concentrations will form part of the final diagnostic algorithm recommended.

### Differences in TG and D-dimers between the African-Caribbean and Caucasian populations

When we compared D-dimer concentrations in women injecting prophylactic doses of enoxaparin, between the African-Caribbean and Caucasian populations, no differences were found. This confirms findings previously reported by our group from a non-pregnant population [7]. Differences in thrombin generation were found between the African-Caribbean women compared to the Caucasian women. These differences were particularly prominent during the first trimester. The last UK confidential enquiry into maternal deaths report [35] reported that 3 of the 5 deaths during the first trimester were of women from an African-Caribbean origin. It could well be, that significantly higher thrombin generation in these women, pre-dispose them to VTE during early pregnancy more so, than the Caucasian population. In part, the increased thrombin generation observed may be related to higher factor VIII levels in the African-Caribbean population [36], and further work is required comparing factor VIII concentrations in these two groups of pregnant women would be useful and might help explain the differences observed.

### Limitations

The results of our work should be considered in the context of their limitations. Not all women had sequential thrombin generation and d-dimer concentrations measured from the first trimester to 8 weeks post-partum. This was largely due to the women booking after the first trimester had passed, and then being referred for haematology input. Furthermore, the sampling of thrombin generation was done at times when women were attending for outpatient appointments; ideally sampling would have been completed in defined trimester ‘windows’, to minimise any impact this may have. Finally, the thrombin generation and ethnicity results were not controlled for weight, which is known to impact significantly on thrombin generation [10]. Future studies specifically assessing thrombin generation differences in different ethnic groups, should be mindful to control for this.

### Future work

There is clear need for a study which measures thrombin generation longitudally during uncomplicated pregnancy. Once the pattern of normal thrombin generation is known, studies can then begin to evaluate if global coagulation assays can detect other pregnancy co-morbidities, e.g. pre-eclampsia or intrauterine growth restriction. Given the differences in TG between the African and Caucasian population, a study formally evaluating TG differences between these two groups would be of value, particularly since the African-Caribbean women represent a significant proportion of women in the last confidential enquires report.

## Conclusion

We found thrombin generation, as measured by CAT, to have increased by the first trimester, with a further increase by the second trimester, after which thrombin generation settled through to delivery. D-dimer concentrations in contrast, increased exponentially during the antenatal period. Both thrombin generation and D-dimers return to baseline levels by 8 weeks postpartum. Our work provides further evidence on the changes of thrombin generation and D-dimers during pregnancy and the puerperium.

## Additional files

Additional file 1: Table S1. Range of enoxaparin doses prescribed for the women recruited to the study. This table illustrates the range of enoxaparin doses prescribed for the women recruited to this study, and the number of women receiving the respective doses. Additional file 2: Table S2. Details on 9 women who were actively managed for VTE during the index pregnancy. This table provides details on the 9 women who were actively managed for thrombosis during the course of this study, and includes the specific indication, the time of presentation and the dose of enoxaparin prescribed. Additional file 3: Table S3. Details on the 10 subjects who were switched from chronic warfarin therapy to enoxaparin during the index pregnancy. This table describes information on 10 subjects who were switched from long-term warfarin to treatment dose enoxaparin whilst they were pregnant. The table provides details on their specific indication for chronic anticoagulation.
