# Supplementary material for: Changes in thrombin generation and D-dimer concentrations in women injecting enoxaparin during pregnancy and the puerperium
Source: BMC Pregnancy Childbirth. 2014 Nov 19;14:384. doi: 10.1186/s12884-014-0384-0 (PMC4240885; doi:10.1186/s12884-014-0384-0)
Supplement: Additional file 1: Table S1. — Range of enoxaparin doses prescribed for the women recruited to the study. This table illustrates the range of enoxaparin doses prescribed for the women recruited to this study, and the number of women receiving the respective doses. [file 12884_2014_384_MOESM1_ESM.docx]

**Supplemental tables (online)**

Table S1: Range of enoxaparin doses prescribed for the women during the study

| Dose of enoxaparin prescribed | *N* | (%) |
| --- | --- | --- |
| 20mg daily | 2 | (2) |
| 40mg daily | 81 | (66) |
| 60mg daily | 2 | (2) |
| 80mg daily | 5 | (4) |
| 100mg daily | 8 | (6) |
| 120mg daily | 3 | (2) |
| 150mg daily | 1 | (1) |
| 180mg daily | 1 | (1) |
| 40mg twice daily | 8 | (6) |
| 50mg twice daily | 1 | (1) |
| 60mg twice daily | 8 | (6) |
| 80mg twice daily | 2 | (2) |
| 100mg twice daily | 1 | (1) |
