# Supplementary material for: Changes in thrombin generation and D-dimer concentrations in women injecting enoxaparin during pregnancy and the puerperium
Source: BMC Pregnancy Childbirth. 2014 Nov 19;14:384. doi: 10.1186/s12884-014-0384-0 (PMC4240885; doi:10.1186/s12884-014-0384-0)
Supplement: Additional file 2: Table S2. — Details on 9 women who were actively managed for VTE during the index pregnancy. This table provides details on the 9 women who were actively managed for thrombosis during the course of this study, and includes the specific indication, the time of presentation and the dose of enoxaparin prescribed. [file 12884_2014_384_MOESM2_ESM.docx]

**Supplemental tables (online)**

Table S2: Details on 9 women who were actively managed for VTE during the index pregnancy

| Subject | Indication | Gestation (weeks) at time of presentation | Enoxaparin dose prescribed |
| --- | --- | --- | --- |
| 17 | DVT (left proximal) | 31 | 100mg daily antenatally and warfarin post-partum |
| 36 | DVT (left proximal) | 23 | 80mg twice a day ante- and postnatally |
| 96 | ^‡^Right long saphenous vein thrombosis | 7 | 80mg daily for six weeks, followed by 40mg daily ante- and postnatally |
| 117 | DVT (left common iliac vein) | 25 | 80mg daily ante- and postnatally |
| 18 | PE (sub-segmental right lower lobe) | 23 | 60mg twice a day ante- and postnatally |
| 44 | PE (extensive bilateral) | 8 | 100mg twice a day ante- and postnatally |
| 49 | PE (VQ probability high) | 23 | 80mg twice a day ante- and postnatally |
| 67 | PE (bilateral sub-segmental) | 22 | 150mg daily ante- and postnatally |
| 79 | Axillary vein thrombosis plus right proximal subclavian vein | 8 | 50mg twice a day antenatally and 80mg once daily postnatally |

‡Not a DVT, but patient was prescribed 6 weeks treatment dose enoxaparin due to venous malformation, following which, this subject was prescribed prophylactic dose enoxaparin for the remainder of her pregnancy and for 6 weeks post-partum.
