# Supplementary material for: Changes in thrombin generation and D-dimer concentrations in women injecting enoxaparin during pregnancy and the puerperium
Source: BMC Pregnancy Childbirth. 2014 Nov 19;14:384. doi: 10.1186/s12884-014-0384-0 (PMC4240885; doi:10.1186/s12884-014-0384-0)
Supplement: Additional file 3: Table S3. — Details on the 10 subjects who were switched from chronic warfarin therapy to enoxaparin during the index pregnancy. This table describes information on 10 subjects who were switched from long-term warfarin to treatment dose enoxaparin whilst they were pregnant. The table provides details on their specific indication for chronic anticoagulation. [file 12884_2014_384_MOESM3_ESM.docx]

**Supplemental tables (online)**

Table S3: Details on the 10 subjects who were switched from warfarin to enoxaparin during the index pregnancy

| Subject | Indication | Target INR range | Enoxaparin dose prescribed |
| --- | --- | --- | --- |
| 16 | Recurrent VTE | 1.5-2.0 | 120mg daily |
| 37 | FVL (homozygous). Symptomatic VTE whilst on combined oral contraceptive pill | 2-3 | 100mg daily |
| 45^†^ | Budd-Chiari (Liver transplant in 1998) | 2-3 | 100mg daily |
| 48 | Paroxysmal nocturnal haemoglobinuria | 2-3 | 80mg daily |
| 53 | Budd-Chiari (Liver transplant in 2006) | 2-3 | 100mg daily |
| 54 | Anti-phospholipid syndrome | 2-3 | 100mg daily |
| 82 | AF/Mitral stenosis/Pulmonary hypertension | 2-3 | 100mg daily |
| 87 | Recurrent VTE | 2-3 | 180mg daily |
| 101^†^ | Budd-Chiari (Liver transplant in 1998) | 2-3 | 100mg daily |
| 115 | Anti-phospholipid syndrome and Budd-Chiari (Liver transplant in 2005) | 3-4 | 60mg daily |

†Same patient
